# Hydrogel Fiber Cultivation Method for Forming Bacterial Cellulose Microspheres

**DOI:** 10.3390/mi9010036

**Published:** 2018-01-17

**Authors:** Kazuhiko Higashi, Norihisa Miki

**Affiliations:** 1 School of Integrated Design Engineering, Keio University, 3-14-1 Hiyoshi, Kohoku-ku, Yokohama 223-8522, Kanagawa, Japan; kazuhiko@z2.keio.jp; 2 Department of Mechanical Engineering, Keio University, 3-14-1 Hiyoshi, Kohoku-ku, Yokohama 223-8522, Kanagawa, Japan

**Keywords:** microbeads, microcarriers, microbes, microfabrication, microfluidics, droplet, emulsion, monodispersity

## Abstract

Forming microspheres or microbeads from nanofibrous materials has recently attracted research interest for their applications in various fields, because these structures greatly impact cellular behaviors and functions. However, conventional methods of preparing microspheres or microbeads have limitations, such as limited variety of material. Here, we propose a new fabrication process for forming a nanofibrous microsphere composed of bacterial cellulose (BC), which is synthesized through fermentation by specific bacteria. The process uses a hydrogel fiber containing spherical cavities. The bacteria encapsulated into the cavities produce BC, resulting in the formation of BC microspheres. Because of its simplicity, robustness, and cost-effectiveness, this process is promising for applications, such as in biochemical engineering and cell delivery systems.

## 1. Introduction

A sphere with a diameter in micrometer range is known as a microsphere, and is widely used in several fields. In biochemical engineering, microspheres provide a large surface area for anchorage-dependent cells. Because of these features, microspheres are referred to as microcarriers, and enable efficient cell culture in large bulk media [1,2,3,4,5,6,7]. These materials can also be used for cell delivery. In recent years, several cells seeded on microspheres have been transplanted to defective organs [8]. Nanofibrous microspheres are promising candidates because of their structural resemblance to the natural extracellular matrix [9,10,11]. Ma et al. synthesized nanofibrous microspheres from star-shaped polymers [12]. However, material options are severely restricted, because the electrospinning method, which is generally used to form nanofibrous materials, is not applicable in microsphere production. Therefore, another method for forming nanofibers is needed.

The natural class of nanofibrous materials known as bacterial cellulose (BC) is produced by specific bacteria such as *Komagataeibacter xylinus*. This bacteria produces cellulosic nanofibers with a diameter of 40–60 nm [13] at a rate of 2 μm/min [14]. Additionally, BC exhibits a high degree of polymerization compared to other celluloses derived from plants; the weight average degree of polymerization of BC is approximately 16,000 [15], exceeding that of cotton (5000) and hardwood pulp (12,000). This enormously high degree of polymerization confers BC with high mechanical and thermal toughness; the fracture stress for elongation of BC is 2.2 MPa [16], and glass-transition temperature *T*_g_ is 300 °C [17]. These values are much higher than those of other natural hydrogels such as agarose, gelatin, and alginate. Moreover, the biocompatibility of BC is well known [18,19]. Biodegradability is a key factor for scaffold applications. Although BC is not enzymatically degradable in vivo, biodegradability can be conferred by oxidizing BC with periodate [20] or 2,2,6,6-tetramethylpiperidine-1-oxyl radical (TEMPO) [21]. Additionally, arginyl-glycyl-aspartic acid (RGD) cell adhesion peptide can be introduced into BC with a xyloglucan-RGD conjugate [22]. Adhesion of human endothelial cells was found to be enhanced compared to native BC. Based on these properties, an increasing number of studies have described tissue engineering employing BC as a scaffold to culture a diverse class of cells ranging from versatile stem cells [23,24,25] to differentiated cells, including smooth muscle cells [26], chondrocytes [27], osteoblasts [28], and cartilage cells [29].

**Figure 1:**
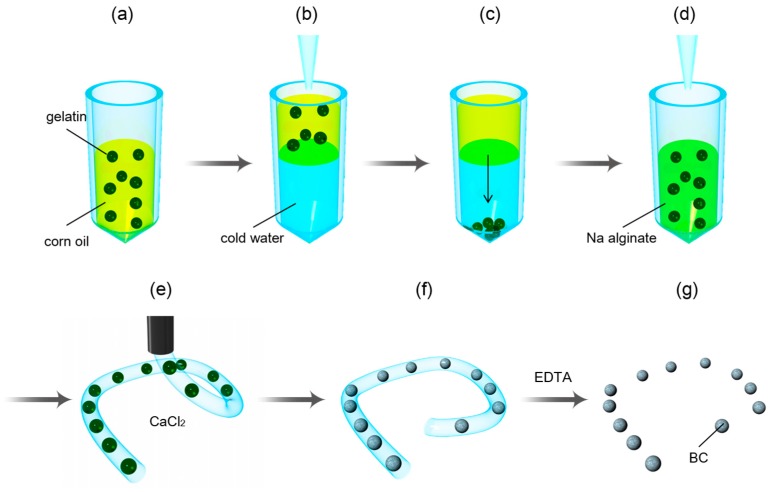
Process flow of the fabrication method: (**a**) Emulsion was cooled to solidify the gelatin containing bacterial cellulose (BC)-producing bacteria. (**b**) The prepared emulsion was placed onto cold water (~2 °C). (**c**) The gelatin gel microspheres in the emulsion were transferred into cold water by centrifugation. (**d**) The gelatin gel microspheres were dispersed in a solution of sodium alginate by changing the dispersion medium. (**e**) A calcium alginate gel fiber containing the gelatin microspheres was formed by casting the sodium alginate solution into calcium chloride solution with a syringe. (**f**) By transferring the formed fiber into the culture medium for the bacteria at 30 °C, the gelatin gel changed to sol state. Here, the bacteria produced BC in the calcium alginate gel fiber. After producing BC, the calcium alginate gel fiber was dissolved in a solution of ethylenediamine tetraacetic acid (EDTA). (**g**) BC microspheres were obtained through this process.

We previously proposed a new process for forming microspheres from BC [30]. This simple process excludes the use of costly materials [31]. In this study, we conducted detailed analyses of the method. As shown in Figure 1, Ca-alginate hydrogel fiber entraps the bacteria into microscopic spherical cavities. The cavities are created using the sol-gel transition of gelatin microspheres containing bacteria. The gelatin microspheres can be prepared by emulsification or microfluidics. In this study, we demonstrated the capability of microsphere preparation from BC by conducting process characterization, feature resolution, microfluidic formation of gelatin sacrificial microspheres, and comparison of the diameters of BC and gelatin microspheres.

## 2. Experimental Section

### 2.1. Materials and Reagents

*Komagataeibacter xylinus* (NBRC 13693) was used to produce BC. All chemicals for cultivating *K. xylinus* including glucose, mannitol, MgSO_4_·7H_2_O, ethanol, polypeptone, and yeast extract, were purchased from Wako Pure Chemical Industries (Osaka, Japan). Deionized water was prepared using a Millipore system (Direct-Q3, Millipore, Billerica, MA, USA). SU-8 (SU-8 10 and 3050) and polydimethylsiloxane (PDMS) (Sylpot 184 W/C) for fabricating PDMS master molds were obtained from MicroChem Corp. (Westborough, MA, USA) and Dow Corning Toray Co., Ltd. (Chiyoda-ku, Japan), respectively. Photomasks for photolithography were manufactured by Tokyo Process Service Co., Ltd. (Tokyo, Japan).

### 2.2. Gelatin Microspheres Preparation by Using Microfluidics

**Figure 2:**
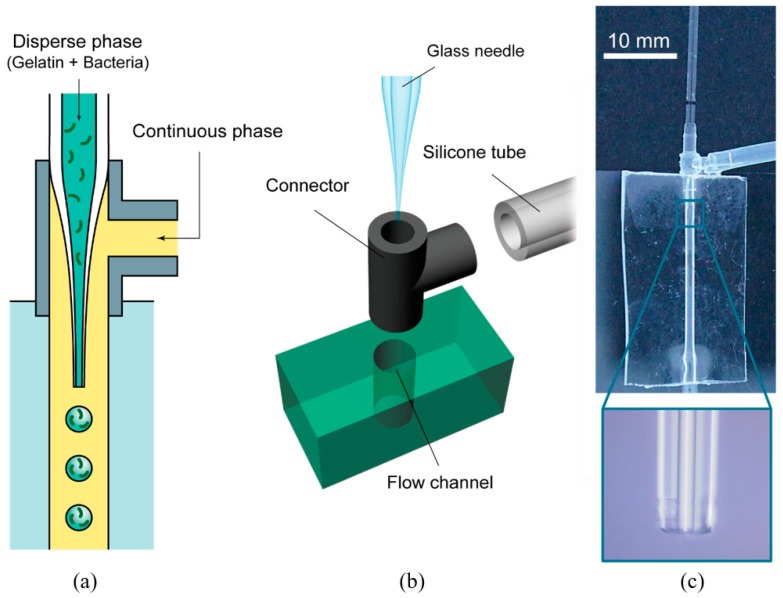
Microfluidic device for generating monodisperse gelatin microspheres. (**a**) The schematic illustration of the microfluidic device. Droplets of the continuous phase were formed at the tip of the glass needle. Prepared emulsion was collected at the end of the glass capillary in cold water. (**b**) The schematic of the microfluidic device composed of a plastic T junction connector, a hollow glass needle, and polydimethylsiloxane (PDMS) flow channel. (**c**) The photographs of the hollow glass needle and the flow channel. The needle was coaxially set in the channel.

To prepare monodisperse gelatin microspheres, a co-flow microfluidic device was used (Figure 2). The microfluidic device was composed of a plastic connector (VTF 106, AS ONE, Osaka, Japan) with an inner diameter of 1.3 mm and hollow glass needle. The device design was similar to those previously reported [32,33,34]; the glass needle was formed by pulling a hollow glass tube (Hirschmann Laboratory, Eberstadt, Germany) using a Puller PC-10 (Narishige Group, Amityville, NY, USA). By introducing a gelatin solution containing *K. xylinus* for the continuous phase and corn oil with span 80 by syringe pumps (AS ONE), droplets were continuously generated at the tip of the glass needle. The droplets were collected in a cold corn oil bath. The mixture of gelatin and bacterial suspension was prepared by mixing the gelatin solution (100 g gelatin, 5 g polypeptone, 5 g yeast extract in 1 L deionized water) and bacterial suspension (gelatin solution: bacterial suspension = 9:1 in volume). For the continuous phase of the emulsion, corn oil containing span 80 (3 wt %) was used. The composition of the culture medium for *K. xylinus* was 5 g polypeptone, 5 g yeast extract, 100 g glucose, 5 g mannitol, 1 g MgSO_4_·7H_2_O, 5 mL ethanol, and 1 L deionized water.

### 2.3. Gelatin Microspheres Preparation by Using Emulsification

The composition of the liquid was the same as that used in the microfluidic method. By stirring a mixture of 1 mL gelatin solution containing *K. xylinus* and 9 mL corn oil containing span 80 with a magnetic stirrer at 750 rpm for 3 min, an emulsion was prepared. In this process, a conical centrifuge tube and magnetic rotor (30 mm in length, 8 mm in diameter) were used.

### 2.4. BC Microspheres Production

The prepared emulsion was poured into cold water (~2 °C) in a plastic tube, followed by centrifugation at 420*g* for 1 min. Subsequently, the gelatin microspheres were transferred into 1.5 wt % Na-alginate solution after removing the corn oil. By extruding the solution into 150 mM CaCCl_2_ solution through a syringe needle, a Ca-alginate hydrogel fiber was formed. The fiber was cultured in the culture medium for *K. xylinus* for 2 days at 30 °C. Inside the cavities, which are created by gelatin microspheres, BC microspheres were produced. The BC microspheres were collected by dissolving the Ca-alginate with 0.5 M ethylenediamine tetraacetic acid (EDTA) solution. The collected BC microspheres were rinsed by autoclaving them with 1 M NaOH for removal of the bacteria.

## 3. Results and Discussion

### 3.1. BC Microspheres Production by Using Microfluidics

**Figure 3:**
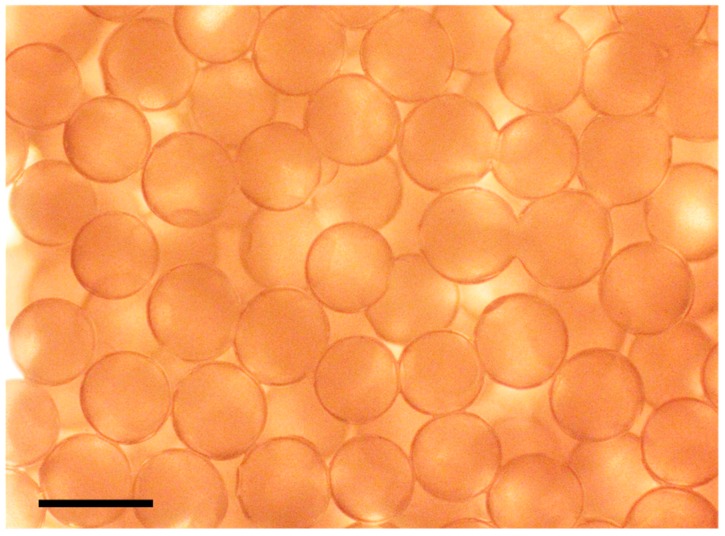
Micrograph of the produced gelatin microspheres using the co-flow microfluidic device. Scale bar, 400 μm.

**Figure 4:**
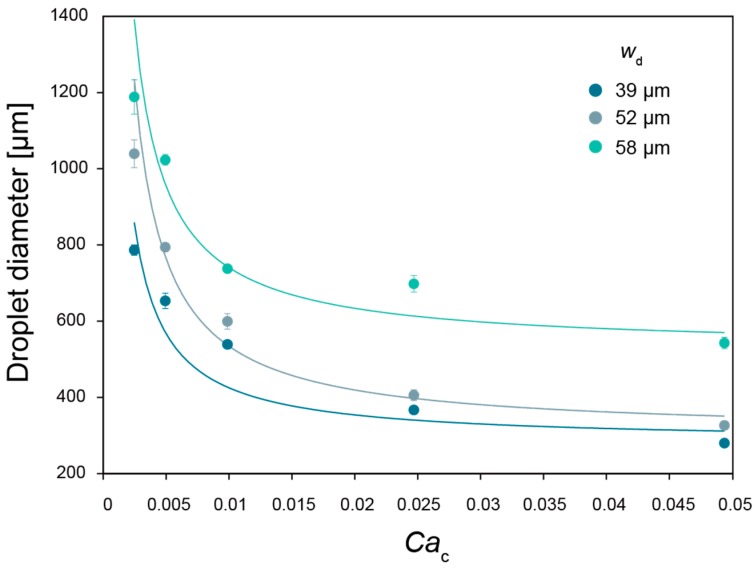
Droplet diameter versus capillary number of the outer fluid for gelatin in corn oil emulsion using the co-flow microfluidic device. Solid curve was a fit using Equation (1), where *w*_d_ = 39 μm, *a* = 7.3, *b* = 0.1; *w*_d_ = 52 μm, *a* = 5.9, *b* = 0.1; *w*_d_ = 58 μm, *a* = 9.1, *b* = 0.1). *Ca*_c_ is computed using the following fluid properties: *η*_c_ = 0.075 Pa s, *γ* = 0.02 N/m, and *w*_c_ = 1.3 mm. Error bars represent standard deviation; sample size *n* > 19.

Figure 3 shows the generated gelatin microspheres by co-flow microfluidic device. In co-flow microfluidic device, there are two modes for the droplet generation: dripping regime and jetting regime. The modes depend on the capillary number of the continuous phase, and the Weber number of the dispersed phase. In this study, droplets were generated only in the dripping regime to achieve high monodispersity. By changing the continuous phase flow rate *q_c_*, or inner diameter of the glass needle *w*_d_, the diameter of the droplet *d* is precisely controllable (Figure 4). In the co-flow device, the diameter ratio $\tilde{d}$ of the droplet is predicted by the following equation [35]:

(1) $$\tilde{d}=a+\frac{b}{3Ca_{c}}$$

where $\tilde{d}=d/w_{d}$ is the dimensionless droplet diameter, $Ca_{c}$ is the capillary number of the continuous phase, and *a*, *b* are fitting parameters. $Ca_{c}$ is expressed as 4*q*_c_*η*_c_/π*w^2^*_c_*γ* by using the volumetric flow rate *q*_c_, viscosity *η*_c_ of continuous phase and interfacial force *γ*, and the flow channel diameter *d*_c_. The solid curves in Figure 3 are fit to Equation (1). From this result of the good agreement of the diameters with the fitting curves, precise control over the droplet diameter can be achieved.

**Figure 5:**
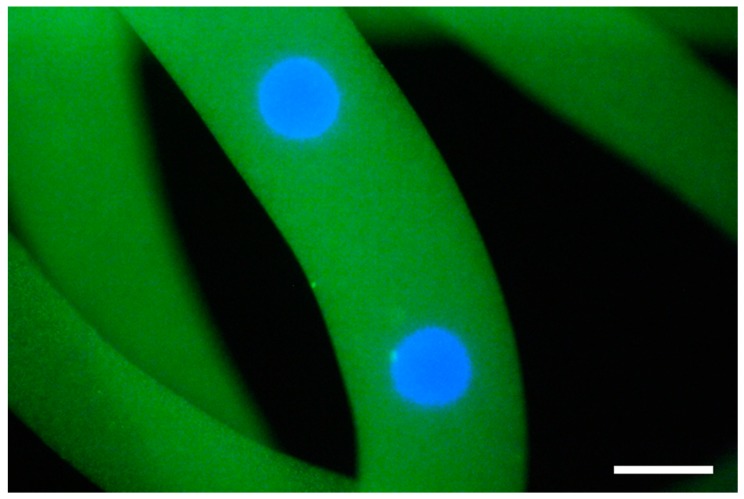
Merged fluorescent micrograph of the Ca-alginate hydrogel fiber containing gelatin microspheres colored with blue and green fluorescent particles. Scale bar, 400 μm.

**Figure 6:**
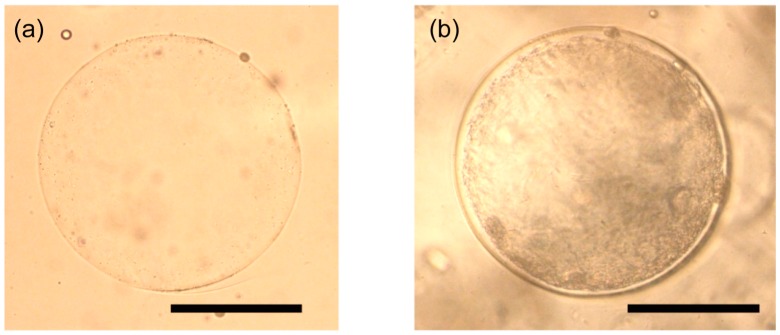
Micrographs of BC microspheres (**a**) at initial state, and (**b**) after 2 day cultivation. Scale bars, 200 μm.

**Figure 7:**
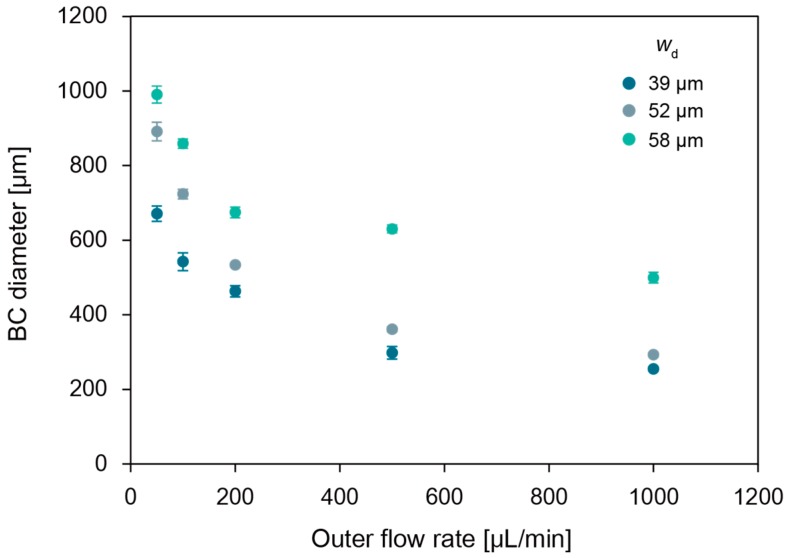
Diameters of BC microspheres using the emulsion prepared from the co-flow microfluidic device. Horizontal axis is the corresponding outer flow rate. Error bars represent the standard deviation; sample size *n* > 19.

**Figure 8:**
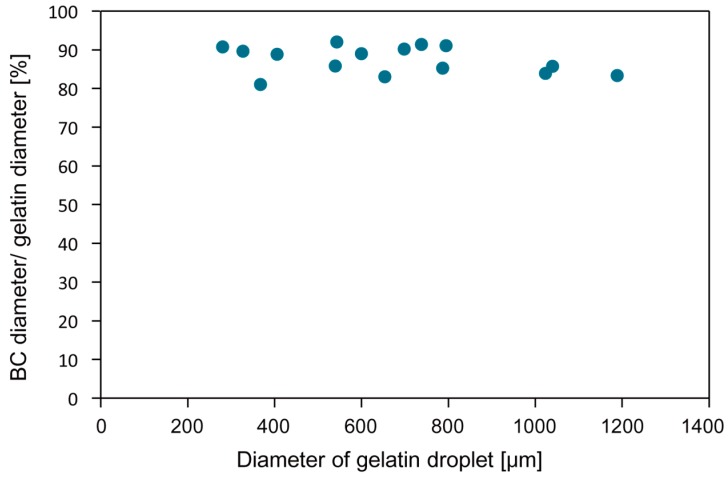
Fraction of BC microsphere diameter to gelatin microsphere diameter as a function of corresponding gelatin microsphere diameter using the microfluidic device.

Figure 5 shows the cavity created inside the Ca-alginate fiber. After 2 day cultivation, a BC microsphere was formed in the cavity (Figure 6b). Figure 7 shows the size of the BC microsphere as a function of corresponding *q*_c_ for gelatin microsphere preparation. The plots show the same tendency as Figure 4. However, the diameters of BC exhibited decrease compared with the ones of gelatin microspheres. As shown in Figure 8, every droplet showed the size decrease of around 15% in diameter. This is probably because of the repulsive force due to the surface charge between the Ca-alginate and the bacteria; the Ca-alginate hydrogel has negative charge on its surface, due to the ionization of the carboxy groups while the surface of the bacterium also has negative charge, due to the ionization of carboxyl groups on the cell membrane [36].

### 3.2. BC Microspheres Production by Using Emulsification

**Figure 9:**
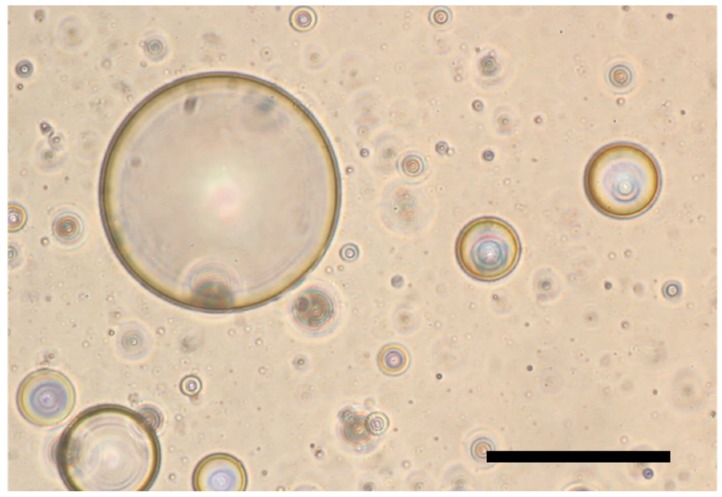
Micrograph of the produced gelatin microspheres using emulsification method. Scale bar, 50 μm.

**Figure 10:**
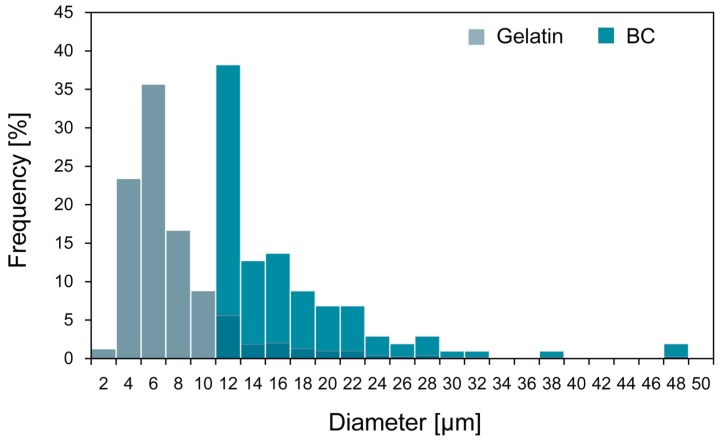
Size distribution of gelatin and BC microspheres using emulsification method. Emulsification was carried out while stirring the two-phase liquids at 750 rpm.

Figure 9 shows the generated gelatin microspheres by emulsification method. BC microspheres formed through emulsification method for preparing gelatin microspheres showed the large distribution in size (Figure 10). For gelatin microspheres, the minimum size of the microsphere was 2 μm. On the other hand, the minimum size of the BC microsphere was 10 μm. This is probably because the minimum space for BC-producing bacteria to form BC microspheres was around 10 μm. The cellulose nanofibers have to be bundled up to form BC. This result implies that the feature resolution of the process is around 10 μm in diameter of the BC microspheres.

## 4. Conclusions

Here, we demonstrated a new process to form BC microspheres by cultivating BC-producing bacteria inside a Ca-alginate hydrogel fiber having spherical cavities created by using gelatin microsphere as a sacrificial architecture. By using an emulsification method to prepare the gelatin microsphere, the minimum diameter of the BC microsphere which the proposed process can form was estimated around 10 μm. Additionally, BC microspheres with high monodispersity were successfully produced by employing a co-flow microfluidic device. Surprisingly, the diameter of the BC was smaller than the one of the cavity in 15%, approximately. These results would be beneficial to the application of the BC microsphere production using the process.
